# Towards a Conceptual Framework for Mechanical Dose in Skeletal Muscle: Integrating Mechanobiology and Resistance Training

**DOI:** 10.3390/jfmk11030368

**Published:** 2026-09-11

**Authors:** Pedro Morouço

**Affiliations:** 1 Department of Sport, Exercise and Health, University of Leiria and Oeste, 2411-901 Leiria, Portugal; pedromorouco@dgs.min-saude.pt; 2 National Program for Physical Activity Promotion, Directorate-General of Health, 1049-005 Lisbon, Portugal; 3 Clínica Espregueira, FIFA Medical Centre of Excellence, 4350-415 Porto, Portugal

**Keywords:** mechanobiology, skeletal muscle, mechanical dose, mechanotransduction, resistance training, muscle adaptation, load monitoring, exercise prescription

## Abstract

Despite major advances in exercise physiology, biomechanics, and mechanobiology, exercise science still lacks a clear conceptual definition of the mechanical stimulus experienced by skeletal muscle during resistance training. Current approaches rely on diverse external, internal, and biomechanical variables (e.g., volume-load, force, power, velocity, time under tension, or muscle architecture), yet none individually captures the muscle-specific mechanical exposure relevant to muscular adaptation. This conceptual inconsistency limits comparisons across studies, complicates training prescription, and hinders the development of individualized monitoring strategies. This integrative review critically synthesizes current evidence from mechanobiology, skeletal muscle physiology, biomechanics, and resistance training to examine how mechanical stimuli are currently conceptualized, quantified, and interpreted. Based on this synthesis, we propose a working definition of Mechanical Dose as the cumulative, muscle-specific mechanical exposure experienced over a defined time window, characterized by loading magnitude, rate, duration, frequency, and spatial distribution, and conditioned by contraction mode and muscle–tendon geometry. Rather than representing a directly measurable variable, mechanical dose is presented as a latent conceptual construct that can only be estimated through combinations of biomechanical, physiological, and morphological indicators. Building upon this definition, we introduce an integrative conceptual framework linking external load, movement biomechanics, mechanical dose, mechanotransduction, and tissue adaptation. We further discuss how this framework may guide future research on the interpretation of field-based monitoring, resistance training prescription, recovery management, and future explainable artificial intelligence approaches in sport science. By reframing mechanical dose as the central construct connecting biomechanics and biological adaptation, this review provides a unified conceptual basis for future research and contributes toward a more biologically informed paradigm of exercise prescription and monitoring.

## 1. Introduction

Resistance training is prescribed through variables that are readily observable and controllable, including external load, repetition number, movement velocity, range of motion, exercise selection, rest intervals, and training frequency [1,2,3]. These variables are fundamental for organizing the training process, yet they do not directly represent the mechanical stimulus experienced by skeletal muscle [4]. The same external load can produce markedly different muscular demands depending on joint configuration, contraction mode, movement strategy, fatigue state, muscle architecture, and individual neuromuscular characteristics [5,6]. Conversely, different training protocols may generate similar muscular adaptations despite substantial differences in their external configuration. This disconnect highlights a persistent conceptual problem in exercise science: training load is routinely quantified, but the biologically effective mechanical stimulus acting on skeletal muscle remains incompletely defined.

The distinction between external and internal load [7] has improved the monitoring of training by separating the work performed from the physiological and perceptual responses elicited by that work [8,9]. However, neither dimension fully captures the mechanical events that initiate muscular adaptation. External-load measures describe what the athlete performs, whereas internal-load measures describe the organism’s psychophysiological response to that work [8]. Mechanical stimuli relevant to skeletal muscle arise at an intermediate level, where external forces are transformed through movement mechanics into muscle-specific forces [10], fascicle deformation, loading rates, contraction durations, and repeated loading cycles [6,11]. These local events are more proximally connected to mechanotransduction than conventional whole-body load metrics, yet they are rarely measured comprehensively in applied settings.

Current resistance-training research therefore relies on multiple partial descriptors of muscular loading. Volume-load, relative intensity, repetition number, time under tension, impulse, mechanical work, power, velocity loss, joint torque, electromyographic amplitude, and estimates of muscle activation [12] are frequently used to characterize the training stimulus [13,14]. More direct approaches, including ultrasonographic assessment of fascicle behavior, elastography, dynamometry, and musculoskeletal modelling, provide additional insight into muscle-specific mechanics [6,15]. Nevertheless, these variables are not interchangeable. Each captures a different component of the loading process, operates at a different biological or mechanical scale, and is subject to distinct assumptions and measurement limitations. Treating any single variable as a complete representation of muscular stimulus risks reducing a multidimensional biological process to an incomplete proxy.

Mechanobiology provides a framework for resolving this conceptual fragmentation. Mechanotransduction encompasses the processes through which cells and tissues convert physical cues into biochemical signals [16,17,18]. Skeletal muscle responds not simply to the presence of load, but to characteristics such as its magnitude, duration, rate, frequency, direction, and spatial distribution [11,19]. Mechanical signals may be sensed through integrin-associated structures, the cytoskeleton, costameres, focal adhesion complexes, mechanosensitive ion channels, the extracellular matrix, and structures associated with the sarcomere, subsequently influencing intracellular pathways involved in protein synthesis [20] and tissue remodeling [21,22]. The resulting biological response is further conditioned by prior loading history, nutritional and metabolic conditions, recovery status, age, training status, and individual responsiveness. Consequently, muscular adaptation cannot be inferred solely from the amount of external work performed.

Despite the centrality of mechanical stimulation to muscular adaptation, the terms mechanical load, mechanical tension, mechanical stimulus, training dose, and mechanical dose are frequently used without a consistent hierarchical or operational distinction [23]. In some resistance-training studies, the presumed mechanical stimulus is represented by external resistance or volume load; in others, it is approximated through force, torque, impulse, work, time under tension, repetition velocity, velocity loss, or muscle activation [11,13]. At the tissue level, related constructs may refer to stress, strain, strain rate, deformation, or cumulative loading cycles. This terminological and measurement heterogeneity limits comparison across studies and obscures the pathway connecting exercise prescription, tissue-level loading, and biological adaptation.

The problem is not merely semantic. Without a coherent definition of mechanical dose [24], it is difficult to determine whether two training protocols deliver equivalent muscle-specific stimuli, whether a monitoring variable has plausible biological meaning, or whether dose–response relationships observed under one exercise configuration can be transferred to another. The absence of a shared framework also constrains individualized prescription. If the stimulus experienced by the muscle cannot be clearly distinguished from the variables used to prescribe or estimate it, the reasoning becomes circular: the prescribed training load is treated as the mechanical dose, while the subsequent adaptation is interpreted as confirmation that the presumed dose was appropriate.

A useful definition must therefore distinguish among four related but non-equivalent levels. First, prescribed load refers to the external variables selected by the practitioner. Second, measured mechanical indicators describe observable or estimated quantities such as force, torque, velocity, impulse, joint kinetics, and fascicle behaviour. Third, Mechanical Dose represents the cumulative muscle-specific mechanical exposure arising from those events over a defined time window. Fourth, mechanotransductive signalling and adaptation are downstream responses. Mechanical Dose is therefore conceptualized as a multidimensional latent construct rather than an outcome inferred retrospectively from whether adaptation occurred [25].

This distinction is particularly relevant in resistance training, where identical program variables may generate different muscle forces and fascicle behaviors across individuals. Anthropometry, exercise technique, joint moment arms, muscle architecture, fatigue, and neural strategy influence how external resistance is transmitted to a particular muscle [6]. Similarly, concentric, eccentric, and isometric actions expose skeletal muscle to different combinations of force, shortening or lengthening velocity, fascicle strain, and contraction duration [5,26]. These differences can result in distinct architectural, molecular, and functional adaptations even when conventional program variables appear comparable [5,27]. A biologically meaningful account of mechanical dose must therefore accommodate both the characteristics of the imposed task and the way the target muscle experiences that task.

The present integrative review addresses this conceptual gap by critically examining how mechanical loading is described across mechanobiology, skeletal muscle physiology, biomechanics, and resistance-training research. Its purpose is not to propose another isolated monitoring metric, but to establish a common conceptual language capable of connecting exercise prescription with tissue-level mechanical events and subsequent adaptation. Specifically, this article aims to: (i) identify the principal constructs currently used to describe muscle-relevant mechanical loading; (ii) clarify the limitations of existing external, internal, and biomechanical descriptors; (iii) propose a working conceptual definition of mechanical dose in skeletal muscle; and (iv) present an integrative framework linking prescribed load, movement biomechanics, mechanical dose, mechanotransduction, and muscular adaptation.

By positioning mechanical dose as the conceptual bridge between training prescription and biological response, the proposed framework is intended to guide the interpretation of resistance-training studies and direct future research toward metrics that are not only measurable, but also mechanistically and biologically meaningful.

## 2. Why Current Concepts Are Insufficient

The variables reviewed below are best understood as partial indicators positioned at different levels of the loading pathway, rather than as competing measures of the same phenomenon. Each provides useful information, but none independently represents the complete muscle-specific mechanical exposure [28].

The distinction between external and internal load has undoubtedly improved the understanding of training responses and remains one of the cornerstones of contemporary load monitoring. External load quantifies the physical work performed by the athlete, whereas internal load reflects the psychophysiological response elicited by that work. However, both constructs remain indirect representations of the stimulus acting upon skeletal muscle. External load describes the demands imposed by the exercise, while internal load reflects the organism’s global response, integrating cardiovascular, metabolic, endocrine, perceptual, and neuromuscular components. Neither construct explicitly characterizes the local mechanical environment experienced by individual muscles or muscle fibers [8,9,29].

To overcome this limitation, biomechanical variables are increasingly incorporated into training monitoring. Joint kinetics, ground reaction forces, barbell velocity, rate of force development, power output, impulse, electromyographic activity, and musculoskeletal modelling all provide a closer approximation of the mechanical conditions under which skeletal muscle operates. Nevertheless, these measures should not be interpreted as equivalent descriptors of muscle loading. Ground reaction force, for example, represents the interaction between the body and the environment rather than the force generated within individual muscles [30]. Likewise, barbell velocity reflects movement performance rather than muscle-specific mechanical conditions, whereas electromyographic amplitude provides information on neural activation but cannot be directly interpreted as a surrogate of muscle force or mechanical tension [6,14,31].

Even variables more directly related to muscle mechanics remain inherently partial. Mechanical work describes energy transfer but does not account for loading rate or temporal distribution [32]. Time under tension reflects contraction duration but ignores force magnitude and spatial heterogeneity [33]. Peak force provides information on maximal loading yet overlooks cumulative exposure, whereas impulse integrates force over time but remains insensitive to the distribution of that force throughout the movement. Similarly, fascicle shortening, pennation angle, tendon behavior, and muscle architecture offer valuable insights into local mechanical behavior but cannot independently describe the overall biological stimulus experienced by the entire muscle [5,11].

A further source of inconsistency arises from the interchangeable use of the terms mechanical load, mechanical stress, mechanical strain, mechanical tension, and mechanical dose [34]. Although these concepts are closely related, they describe different physical or biological phenomena. Mechanical load generally refers to the externally applied force or resistance. Mechanical stress represents the internal force normalized to tissue area, whereas strain describes tissue deformation relative to its original dimensions. Mechanical tension is commonly used within resistance training to describe the force transmitted through muscle fibers, although its definition varies considerably across studies. Mechanical dose, in contrast, has rarely been explicitly defined and is often implicitly substituted by one of the preceding variables. Consequently, identical terminology may refer to substantially different constructs depending on the disciplinary perspective adopted [35], creating ambiguity when interpreting both experimental findings and practical recommendations.

This conceptual ambiguity has important methodological consequences. Studies investigating ostensibly similar interventions frequently quantify the training stimulus using entirely different variables, making direct comparison difficult and limiting the synthesis of evidence across the literature. More importantly, the absence of a common conceptual framework hinders the interpretation of dose–response relationships. When muscle hypertrophy, architectural remodeling, or strength gains are observed following resistance training, it remains unclear whether these adaptations are primarily associated with external load, contraction-specific mechanics, cumulative force exposure, local tissue deformation, or a combination of these factors. Consequently, much of the existing literature evaluates surrogate measures of the mechanical stimulus without explicitly distinguishing between what is directly measured and what is biologically inferred [6].

This distinction becomes increasingly relevant as wearable technologies, musculoskeletal models, and artificial intelligence are incorporated into athlete monitoring. Modern systems can generate an unprecedented volume of biomechanical information; however, greater data availability does not necessarily imply improved biological interpretation [36]. Predictive models remain constrained by the quality and physiological relevance of their input variables. If the construct being estimated lacks a clear conceptual definition, increasingly sophisticated analytical methods merely provide more precise estimates of an ill-defined target [37]. As highlighted in recent discussions on injury prediction and athlete monitoring, improving prediction requires not only better algorithms but also more meaningful representations of the underlying biological processes [36,38].

Taken together, these observations suggest that the principal limitation is not the absence of measurable variables, but rather the absence of an integrative framework capable of positioning each variable according to its biological significance. External load, biomechanical indicators, and physiological responses should therefore be viewed as complementary sources of information rather than competing representations of the same construct. From this perspective, mechanical dose should not be interpreted as another measurable variable to be added to existing monitoring systems. Instead, it should be regarded as a higher-order latent construct integrating the magnitude, rate, duration, frequency, and spatial distribution of mechanical exposure experienced by a target muscle.

## 3. Mechanobiology as the Biological Foundation of Mechanical Dose

Mechanical loading represents the primary environmental stimulus governing skeletal muscle adaptation. Nevertheless, muscles do not respond directly to externally applied forces or prescribed training variables. Instead, adaptation emerges from the conversion of mechanical perturbations into intracellular biochemical signals through a process collectively known as mechanotransduction [18,39]. From this perspective, the biological relevance of any training stimulus is determined not by the amount of weight lifted or the number of repetitions performed, but by how mechanical information is sensed, integrated, and interpreted by muscle tissue.

Mechanotransduction is increasingly recognized as a multiscale process extending from whole-body movement to molecular signaling. External forces generated during resistance exercise are first transformed into joint kinetics and muscle–tendon mechanics before producing local tissue deformation at the level of muscle fibers and the extracellular matrix [40]. These deformations are subsequently detected by specialized mechanosensitive structures (e.g., integrin-based focal adhesions, costameres, the dystrophin-associated glycoprotein complex, mechanosensitive ion channels, and cytoskeleton) [41], which convert physical stimuli into intracellular signaling cascades regulating protein synthesis, extracellular matrix remodeling, satellite cell activation, and ultimately muscle adaptation [11,16,21].

Importantly, mechanotransduction is not triggered by a single mechanical variable. Experimental evidence consistently demonstrates that skeletal muscle cells respond to the interaction of multiple characteristics of the applied stimulus, including loading magnitude, contraction duration, strain amplitude, strain rate, loading frequency, temporal distribution, and recovery intervals [11,19,42]. Consequently, the biological response reflects the cumulative integration of several mechanical dimensions rather than the isolated influence of force, velocity, or work alone [17]. This observation provides one of the strongest biological arguments against interpreting any single biomechanical metric as a complete representation of the stimulus experienced by skeletal muscle.

Mechanotransductive pathways also exhibit different temporal kinetics: mechanosensing and phosphorylation events may occur during or shortly after loading, whereas transcriptional regulation, protein turnover, extracellular-matrix remodelling, and structural adaptation develop over longer periods. Consequently, the timing of biological sampling must be specified when candidate Mechanical Dose models are evaluated.

Furthermore, identical external mechanical conditions do not necessarily result in equivalent biological responses. Muscle architecture, fascicle length, pennation angle, tendon compliance, neural recruitment strategies, fiber-type composition, previous loading history, metabolic state, age, sex, nutritional status, and genetic factors [43,44] all influence how a given mechanical stimulus is distributed throughout the tissue and how effectively it activates mechanosensitive pathways [5,22]. Consequently, mechanical stimuli should not be viewed as absolute quantities but rather as context-dependent biological signals whose effectiveness depends on tissue-specific responsiveness.

Resistance training provides a particularly clear illustration of this principle. Concentric, eccentric, and isometric contractions may be prescribed using similar external loads while exposing skeletal muscle to markedly different combinations of fiber shortening or lengthening, fascicle behavior, force production, contraction duration, and mechanical strain [45]. These distinct mechanical environments activate overlapping but non-identical signaling pathways, contributing to differences in muscle architecture, regional hypertrophy, connective tissue remodeling, and functional performance [5,26,27,46]. Likewise, two individuals performing the same exercise with identical external resistance may experience substantially different internal mechanical environments because of differences in anthropometry, lifting technique, neuromuscular coordination, or fatigue. These observations reinforce the notion that externally prescribed load should not be interpreted as synonymous with the biologically effective stimulus.

From a mechanobiological perspective, adaptation therefore depends less on isolated mechanical variables than on the integrated mechanical history experienced by the tissue [47]. Rather than responding exclusively to peak loading events, skeletal muscle appears to integrate the cumulative characteristics of repeated loading cycles over time. Magnitude, duration, loading rate, frequency, recovery periods, and spatial distribution collectively determine the extent to which mechanosensitive pathways are activated and whether the resulting adaptation favors hypertrophy, architectural remodeling, maintenance, or maladaptation [11,21,48]. This cumulative interpretation aligns with emerging concepts in systems biology, where biological responses are increasingly understood as the consequence of integrated signals rather than isolated stimuli.

Another important implication concerns the distinction between mechanical exposure and mechanical effectiveness [23]. Two exercises may generate comparable external forces while producing markedly different cellular responses because the mechanical stimulus is distributed differently across muscle fibers, connective tissue, and the extracellular matrix. Similarly, variables commonly monitored in applied settings (e.g., barbell velocity, force production, repetition number, power output) describe mechanical exposure but not necessarily the biological effectiveness of that exposure [49]. Their interpretation therefore requires an explicit conceptual framework linking observable biomechanical variables with tissue-level mechanobiology.

Collectively, current mechanobiological evidence suggests that mechanical load alone is insufficient to characterize the multidimensional exposure experienced by skeletal muscle. In the present framework, Mechanical Dose describes cumulative exposure, whereas mechanotransductive signalling and adaptation are treated as downstream responses moderated by tissue responsiveness [50].

## 4. Towards a Conceptual Framework for Mechanical Dose

As previously mentioned, defining mechanical dose as any single measurable quantity inevitably oversimplifies the biological process underlying muscular adaptation [23]. Thus, we propose that mechanical dose should be conceptualized as a latent conceptual construct rather than a directly measurable biomechanical variable [25]. Like concepts such as cardiovascular fitness, frailty, or biological age, mechanical dose cannot be observed directly. Instead, it must be inferred from multiple measurable indicators that collectively describe the mechanical environment experienced by skeletal muscle [36,38].

Mechanical loading in resistance training is currently described through multiple partial indicators [11,13,36,38]. The present contribution is therefore framed as a skeletal-muscle-specific conceptual synthesis rather than as a claim that mechanical-dose concepts are entirely new. Accordingly, we propose the following working definition: Mechanical Dose is the cumulative, muscle-specific mechanical exposure experienced over a defined time window, characterized by the magnitude, rate, duration, frequency, and spatial distribution of tissue-level loading, and conditioned by contraction mode and muscle–tendon geometry. Mechanotransductive signalling and subsequent adaptation are downstream responses and are not part of the dose definition; tissue responsiveness moderates the dose–response relationship rather than defining the dose.

**Figure 1:**
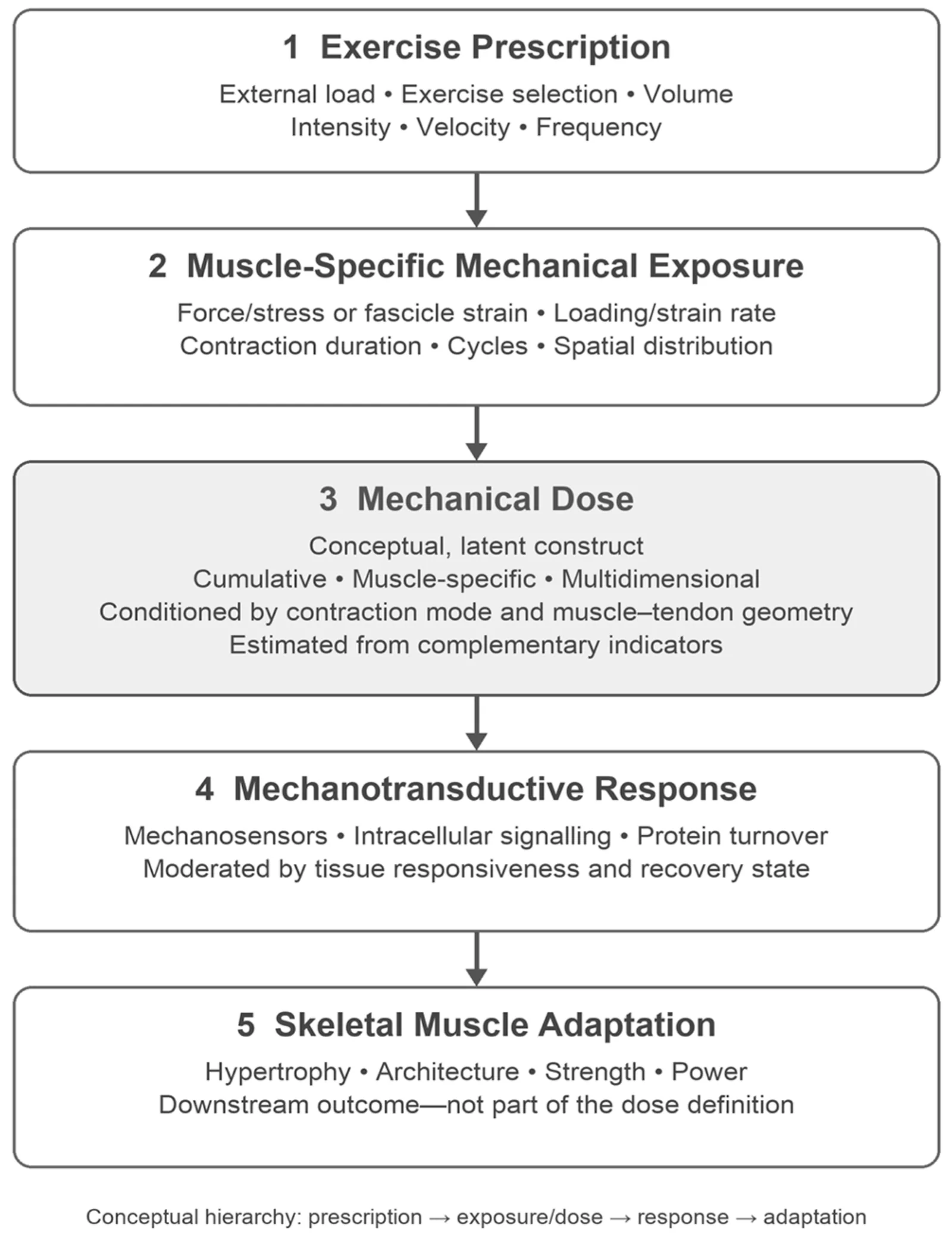
Conceptual hierarchy underlying the proposed framework for Mechanical Dose. Exercise prescription generates measurable or estimated muscle-specific mechanical exposure. Mechanical Dose integrates the cumulative exposure profile across magnitude, rate, duration, frequency, and spatial distribution, conditioned by contraction mode and muscle–tendon geometry. Mechanotransductive signalling and muscular adaptation are downstream responses, while tissue responsiveness moderates the dose–response relationship rather than defining the dose.

The conceptual relationships are summarized in Figure 1, which separates exercise prescription, measurable mechanical exposure, Mechanical Dose, mechanotransductive response, and subsequent adaptation.

First, mechanical dose is cumulative. Muscular adaptation rarely results from a single loading event but rather from the repeated integration of mechanical stimuli across successive contractions and training sessions [11,21]. Peak forces may contribute to adaptation, yet their biological significance depends on the broader loading history within which they occur.

Second, Mechanical Dose is exposure-based rather than outcome-defined. Mechanical events are characterized by how they are transmitted to and distributed within the target tissue [16,17]. Consequently, two mechanically similar exercises may produce different exposure profiles because of local tissue mechanics, muscle architecture, or neuromuscular recruitment [5]. Whether either profile produces greater adaptation remains a separate empirical question.

Third, mechanical dose is muscle-specific. Skeletal muscles differ in architecture, fiber-type composition, tendon compliance, moment arms, neural recruitment strategies, previous loading history and adaptive capacity [5,43]. Therefore, the same external exercise cannot be assumed to produce identical mechanical doses across muscles or individuals [51]. Mechanical dose should always be interpreted relative to the tissue of interest rather than to the exercise itself.

Fourth, mechanical dose is multidimensional. Rather than being determined exclusively by loading magnitude, it emerges from the interaction of multiple mechanical dimensions, including force, contraction duration, loading rate, strain characteristics, contraction mode, frequency of exposure, recovery intervals, and spatial distribution of mechanical stimuli [11,19]. These dimensions are not additive but interactive, meaning that changes in one characteristic may modify the biological significance of another [42].

Finally, mechanical dose is estimated rather than measured [15]. Current technologies (e.g., force platforms, dynamometry, velocity-based devices, wearable sensors, ultrasonography, and electromyography) provide valuable but incomplete information regarding the mechanical environment of skeletal muscle [6,14,52]. Each captures a different aspect of the underlying construct. Consequently, the purpose of future monitoring systems should not be to identify a single “best” metric but to integrate complementary sources of information capable of improving estimation of the latent construct represented by Mechanical Dose [36,38].

For conceptual purposes, a dimensionless Mechanical Dose index for muscle m over time window T could be written as follows:

MD*m(T) = Σ_i=1_^N(T)^ W(Δt_i_) (M_m,i_/Mref)^α^ (R_m,i_/Rref)^β^ (D_m,i_/Dref)^γ^ S_m,i_ C_m,i_

Here, M is a muscle-specific loading-magnitude indicator (for example, estimated muscle force or stress or fascicle strain), R is the loading or strain rate, D is the contraction duration, N(T) is the number of loading events, W(Δt) represents the temporal spacing of weights and recovery between events, S represents spatial distribution, and C modifies the contribution according to contraction mode and muscle–tendon geometry. Reference values render the terms dimensionless. The exponents α, β, and γ are intentionally unspecified. This equation is an illustrative conceptual scaffold, not a validated equation, final metric, or claim that the dimensions combine multiplicatively. Competing functional forms should be compared prospectively.

This distinction also has important methodological implications. Much of the current debate surrounding training monitoring implicitly assumes that the optimal variable remains to be discovered. From the perspective proposed here, however, this assumption may be fundamentally misplaced. The objective should not be to replace one proxy with another, but rather to develop biologically informed models capable of integrating multiple indicators according to their physiological relevance [36,37]. In this context, advances in wearable sensing, musculoskeletal modelling, digital twins, and explainable artificial intelligence should be viewed as complementary tools for improving estimation of mechanical dose rather than as direct measures of the construct itself [38].

Importantly, the proposed definition does not replace existing biomechanical variables. External load defines the exercise challenge; biomechanical indicators describe how that challenge is expressed; their cumulative muscle-specific integration constitutes Mechanical Dose; and mechanobiological processes describe the downstream response [8,11]. This hierarchy may reconcile constructs that have often been treated as competing descriptors.

Collectively, this conceptual definition shifts the emphasis from identifying a single optimal monitoring variable towards understanding how multiple complementary measurements converge to estimate the muscle-specific mechanical exposure associated with downstream skeletal muscle responses. In doing so, it establishes a conceptual bridge between biomechanics, mechanobiology, and resistance-training prescription that can serve as a common framework for both future research and applied practice.

## 5. Translating Mechanical Dose into Resistance Training Practice

The conceptual definition proposed in this review is only useful if it provides a framework capable of improving exercise prescription and monitoring. Rather than introducing another training variable, the concept of mechanical dose offers a different perspective for interpreting existing measurements. It encourages practitioners to move beyond the search for a single “best” monitoring metric and instead consider how multiple complementary variables collectively describe the muscle-specific mechanical exposure experienced by skeletal muscle [7]. From this perspective, exercise prescription becomes less focused on reproducing identical external loads and more focused on reproducing comparable Mechanical Dose profiles [1].

### 5.1. Mechanical Dose as a Multidimensional Construct

Consider two sessions performed by the same individual using the same exercise and matched for conventional volume-load. A heavy, low-repetition condition may produce greater muscle-force magnitude but fewer loading cycles and a shorter cumulative contraction duration [5,11]. A moderate-load condition performed with more repetitions or longer contractions may produce lower peak magnitude but greater cumulative duration and a different loading- or strain-rate profile [32,49].

Conventional volume load would classify the sessions as equivalent [53], whereas the proposed framework characterizes them as distinct Mechanical Dose profiles because contraction velocity, muscle length, and loading distribution may differ [5,13]. Importantly, the framework does not infer which profile will produce superior adaptation; that relationship remains an empirical question.

In practice, the user would specify the target muscle and observation window, and then select feasible indicators for the relevant dimensions. External load and repetition data could be combined with force–time or velocity measures and, where feasible, muscle-specific modelling or imaging [8,11,28]. The resulting estimate should retain uncertainty and remain distinct from the biological response observed afterwards.

### 5.2. Implications for Monitoring Skeletal Muscle Adaptation

Current athlete-monitoring systems frequently combine measures of external load, performance, and subjective responses. Although this multidimensional approach has substantially improved decision-making, interpretation often remains centered on isolated variables rather than on their biological interaction [9,36]. The proposed concept of mechanical dose suggests that monitoring should focus less on identifying a single optimal metric and more on integrating complementary measurements that collectively improve estimation of the tissue-specific mechanical environment.

Variables such as force–time characteristics, movement velocity, power output, fascicle behavior, muscle architecture, and neuromuscular performance should therefore be interpreted as different windows into the same underlying construct rather than as independent indicators of adaptation [6,14,54]. Equally important is recognition that the biological meaning of each variable depends on the exercise performed, the muscle being evaluated, and the specific adaptation of interest [55].

This interpretation is particularly relevant for emerging wearable technologies and musculoskeletal modelling [52]. Advances in sensing technologies have substantially increased the quantity of biomechanical information available to practitioners. However, improvements in data acquisition do not necessarily translate into improvements in biological understanding. Without a clear conceptual target, increasing data complexity risks generating greater analytical precision without improving physiological interpretation [37,38].

### 5.3. Towards Individualized Exercise Prescription

Perhaps the greatest contribution of the proposed framework lies in its implications for individualized resistance training. Mechanical dose is unlikely to be identical across individuals performing the same exercise because the effective stimulus depends on factors including muscle architecture, neuromuscular coordination, fatigue, previous training exposure, recovery status, and adaptive capacity [5,22,33]. Consequently, prescribing identical external loads should not be interpreted as prescribing identical biological stimuli.

Future resistance-training prescription may benefit from integrating biomechanical measurements, physiological monitoring, musculoskeletal modelling, wearable or instrumented-device data, and longitudinal training and recovery information within biologically informed decision-support systems [15,36]. Machine-learning models may help integrate interacting variables, whereas large language models could provide a practitioner-facing interface for explaining model outputs and supporting real-time adjustments [38]. At present, however, these tools should be assistive rather than autonomous and remain subject to professional oversight, validation, and privacy safeguards.

Ultimately, the practical value of the proposed framework does not depend on introducing new monitoring devices or replacing existing training variables. Its principal contribution is conceptual: providing a biologically grounded framework that explains how existing measurements should be interpreted collectively rather than individually [8]. This shift in perspective may facilitate more coherent exercise prescription, improve interpretation of athlete-monitoring data, and guide the development of future methodologies explicitly designed to estimate the muscle-specific mechanical exposure experienced by skeletal muscle.

## 6. Conclusions

Resistance training has traditionally been prescribed and monitored through variables that describe either the external demands imposed by exercise or the physiological responses elicited by those demands. Although these approaches have substantially advanced both research and applied practice, they do not explicitly define the muscle-specific mechanical exposure experienced by skeletal muscle. As a result, concepts such as external load, mechanical tension, work, power, force, time under tension, and muscle activation have frequently been used as interchangeable descriptors of a phenomenon that is, in reality, multidimensional and biologically complex.

The present review argues that this conceptual ambiguity limits both scientific interpretation and practical application. Rather than representing equivalent measures of the same construct, current biomechanical and physiological variables each capture different components of the mechanical environment acting upon skeletal muscle. Their apparent inconsistencies should therefore not be interpreted as methodological shortcomings, but as evidence that no single measurement can fully characterize the biological stimulus responsible for muscular adaptation.

Drawing upon current knowledge in mechanobiology, skeletal muscle physiology, biomechanics, and resistance training, we propose that Mechanical Dose should be understood as cumulative, muscle-specific mechanical exposure over a defined time window. It is a latent conceptual construct, not a directly measurable variable, and mechanotransductive signalling and adaptation are downstream responses rather than components of its definition.

This perspective shifts the emphasis of resistance-training research from identifying an optimal individual metric towards developing biologically informed models capable of integrating complementary sources of information. External load, biomechanical measurements, Mechanical Dose, mechanotransductive responses, and adaptation should therefore be viewed as successive levels within a common hierarchy rather than as competing explanations. Such an interpretation offers a coherent framework capable of linking exercise prescription with the cellular and tissue-level mechanisms that ultimately determine muscular adaptation.

The proposed framework also provides a research agenda for the next generation of resistance-training studies. Future investigations should move beyond validating isolated biomechanical variables and instead focus on determining how combinations of mechanical indicators relate to mechanotransductive signaling, architectural remodeling, hypertrophy, strength development, and long-term functional adaptation. Particular emphasis should be placed on longitudinal human studies integrating advanced biomechanical assessment, musculoskeletal modelling, imaging techniques, and molecular biomarkers. These approaches will be essential to establish whether the proposed construct of mechanical dose can be operationalized with sufficient precision for routine application in both research and practice.

From an applied perspective, the concept of mechanical dose encourages practitioners to reconsider the objective of exercise prescription. Rather than attempting to reproduce identical external loads across athletes, coaches and clinicians should aim to reproduce comparable Mechanical Dose profiles, recognizing that these are inherently influenced by individual morphology, neuromuscular function, training history, and adaptive capacity. Although direct quantification of mechanical dose remains beyond current technological capabilities, recent advances in wearable sensing, computational biomechanics, digital twins, and explainable artificial intelligence provide promising opportunities to progressively improve its estimation.

Ultimately, the contribution of this review lies not in introducing another monitoring variable, but in proposing a common conceptual language capable of integrating biomechanics, mechanobiology, and resistance-training science. If validated through future experimental research, the concept of mechanical dose may provide the missing theoretical link between what practitioners prescribe, what skeletal muscle experiences, and how adaptation ultimately occurs.

## Data Availability

No new data were created or analyzed in this study. Data sharing is not applicable to this article.
